# PET/MRI in large-vessel vasculitis: clinical value for diagnosis and assessment of disease activity

**DOI:** 10.1038/s41598-019-48709-w

**Published:** 2019-08-27

**Authors:** Charlotte Laurent, Laure Ricard, Olivier Fain, Irene Buvat, Amir Adedjouma, Michael Soussan, Arsène Mekinian

**Affiliations:** 1 AP-HP, Sorbonne Université, Hôpital Saint-Antoine, Service de Médecine Interne and Inflammation-Immunopathology-Biotherapy Department (DHU i2B), F-75012 Paris, France; 2 IMIV, CEA, INSERM, Université Paris Sud, CNRS, Université Paris Saclay, Orsay, France; 3 APHP, Hôpital Avicenne, Service de Médecine Nucléaire, Paris 13 University, Bobigny, France

**Keywords:** Diseases, Autoimmune diseases, Outcomes research

## Abstract

Diagnosis of large vessel vasculitis (LVV) and evaluation of its inflammatory activity can be challenging. Our aim was to investigate the value of hybrid positron-emission tomography/magnetic resonance imaging (PET/MRI) in LVV. All consecutive patients with LVV from the Department of Internal Medicine who underwent PET/MRI were included. Three PET/MRI patterns were defined: (i) “inflammatory,” with positive PET (>liver uptake) and abnormal MRI (stenosis and/or wall thickening); (ii) “fibrous”, negative PET (≤liver uptake) and abnormal MRI; and (iii) “normal”. Thirteen patients (10 female; median age: 67-years [range: 23–87]) underwent 18 PET/MRI scans. PET/MRI was performed at diagnosis (n = 4), at relapse (n = 7), or during remission (n = 7). Among the 18 scans, eight (44%) showed an inflammatory pattern and three (17%) a fibrous pattern; the other seven were normal. The distribution of the three patterns did not differ between patients with Takayasu arteritis (TA, n = 10 scans) and those with giant cell arteritis (GCA, n = 8 scans). PET/MRI findings were normal in 2/10 (20%) TA scans vs. 5/8 (62%) GCA scans (p = 0.3). Median SUV_max_ was 4.7 [2.1–8.6] vs. 2 [1.8–2.6] in patients with active disease vs. remission, respectively (p = 0.003). PET/MRI is a new hybrid imaging modality allowing comprehensive and multimodal analysis of vascular wall inflammation and the vascular lumen. This technique offers promising perspectives for the diagnosis and monitoring of LVV.

## Introduction

Large-vessel vasculitis (LVV) is defined as a vasculitis affecting large arteries of which giant cell arteritis (GCA) and Takayasu’s arteritis (TA) represent the two main forms^1^. LVV is characterized by arteritis of the aorta and its main branches which can lead to stenosis and aneurysms. The diagnosis of GCA can be challenging, particularly in patients with symptoms consistent with GCA but negative temporal artery biopsy (TAB). In these patients, it is necessary to demonstrate arterial involvement in order to confirm the diagnosis of GCA. In TA, the assessment of disease activity can be challenging and positron-emission tomography (PET)-imaging can be useful to distinguish fibrotic stenosis from active arterial lesions.

Several studies have shown good sensitivity of fluorine-18-fluorodeoxyglucose (FDG)-PET/computed tomography (CT) for the diagnosis of arterial involvement in LVV, with a sensitivity around 89.5% for GCA and 87% for TA^2–6^. In a recent double-blinded study of 64 newly suspected GCA, the sensitivity and specificity of PET/CT were 92% and 85% respectively compared with TAB, with high negative predictive value^7,8^. In TA, a recent study in twenty-six patients who underwent graft surgery have shown that significant 18F- FDG uptake that is confined to arterial graft sites does not reflect clinically relevant disease activity or progression^9^. Available evidence supports the use of PET/magnetic resonance imaging (MRI) on clinic routine and research^10,11^, with a strong focus on oncology^12^. Very few studies have focused on FDG-PET/MRI in LVV. Among 12 patients who underwent PET/MRI, maximum standardized uptake value (SUVmax) and visual scores did not differ between PET/MRI and PET/CT^13^. In a recent retrospective study, 14 patients with aortitis and 14 patients without aortitis underwent 18F-FDG for the evaluation of inflammatory aortic involvement. All patients were imaged at 3 T using T1W VIBE pre- and post-contrast in order to compare these two imaging techniques. The results demonstrate that T1W VIBE MRI of the aorta detects vessel wall inflammation in a comparable number of patients with LVV in relation to 18F-FDG PET^14^. Therefore, the use of PET/MRI for LVV diagnosis and follow-up remains to be determined.

The aim of this study was to describe the PET/MRI findings in patients with LVV and to determine their relation with clinical outcome.

## Patients and Methods

### Patients

The study included patients with LVV (TA and GCA) from the University Department of Internal Medicine, Saint-Antoine hospital, who underwent PET/MRI between October 2015 and June 2017. Inclusion criteria were: diagnosis of TA, with Ishikawa modified Sharma criteria^15,16^, or GCA with American College of Rheumatology criteria^17^; PET/MRI at any time during the disease course, and the absence of infectious complications or known neoplasia at the time of PET/MRI. All consecutive patients with LVV have been proposed to participate to this study.

Clinical and laboratory data and treatments were analysed at LVV diagnosis, at the time of PET/MRI, and at the last evaluation. The presence of constitutional symptoms and vascular impairment (dizziness, visual disturbances, faint or absent pulse, differences in systolic blood pressure between the arms) were recorded at each evaluation. C-reactive protein (CRP) levels were recorded. For patients with ongoing immunosuppressive therapy, data on steroid and other immunosuppressive therapies were obtained at disease diagnosis and at the time of PET/MRI. For GCA, active disease was defined as the presence of clinical signs and increased CRP level (>10 mg/L) and for TA, a National Institutes of Health stroke score >2. All patients were enrolled prospectively in the “Promise” protocol (2015-A01431-48) and a written informed consent was obtained from all patients. This study was approved by national government authorities (CPP Ile de France, Ambroise Paré, Paris France N° 2015-A01431-48).

### FDG-PET/MRI protocol and analysis

PET/MRI scans were performed with a hybrid system (Signa PET/MRI; General Electric) enabling simultaneous acquisition of PET and 3 Tesla (3 T)-MRI images (axial field view: 25.0 cm; transverse field view: 60 cm; spatial resolution at a radial distance of 1 cm: 4.3 mm (transverse) x 5.3 mm (axial); sensitivity: 23.3 cps/kBq). PET/MRI data were acquired a median of 120 min after intravenous injection of FDG (4 to 5 MBq/kg), and were obtained from the skull to the mid-thigh, involving 5–7 bed positions depending on the patient’s height, with 3 min/bed position. Serum glucose levels before FDG administration were under 1,4 g/L. A respiratory bellow was used for respiratory triggering of MRI acquisitions. PET images were reconstructed using OSEM (two iterations/28 subsets) with time-of-flight and point spread function (PSF) modelling. Results were displayed in a 192 × 192 matrix with 3 × 3 × 2.8 mm^3^ voxels. Reconstructed images were post-filtered by Gaussian filtering with full width at half maximum of 5 mm. The MRI protocol involved four sequences: (i) head to mid-thigh Dixon acquisition for MRI-based attenuation correction (repetition time: 4 ms; echo time: 1.7 ms; flip angle 5°; 256 × 128 matrix); (ii) head to mid-thigh axial T2 fast recovery fast spin echo sequence (TR/TE 4000/102 ms; flip angle 111°; 320 × 256 matrix; two signals acquired) with respiratory triggering covering the abdomen and thorax to detect vessel wall edema; (iii);. Coronal 3D fat suppressed MR angiography, using a fast spoiled gradient echo recalled (SPGR) sequence, acquired 10 sec after injection of contrast medium (TR/TE/ 3.7/1.2 ms, flip angle 30°, matrix 320 × 192, slice thickness of 2.8 mm). Post-processing was performed using maximum intensity projection (MIP); and (iv) breath-hold contrast-enhanced 3D T1-weighted sequence (LAVA FLEX, TR/TE 5.5/1.7 ms; flip angle 15°; 320 × 256 matrix; one signal acquired) covering the neck, thorax, abdomen, and pelvis to detect vessel wall thickening and enhancement by contrast medium. Contrast medium (gadoretic acid 0.5 mmol/ml) was injected intravenously at a standard dose of 0.2 mL/kg body weight.

### Image analysis

Visual classification of vascular uptake was carried out, as recommended in recent recommendation^18^: 0 = no uptake (≤mediastinum); 1 = low-grade uptake (<liver); 2 = intermediate-grade uptake (=liver), 3 = high-grade uptake (>liver), with grade 3 considered positive for active LVV. Eleven arterial segments were analyzed: four aortic segments (ascending thoracic aorta, aortic arch, descending thoracic aorta, and abdominal aorta), right and left common carotid arteries, right and left subclavian arteries, vertebral arteries, and common iliac and femoral arteries. PET images were interpreted by a resident and reviewed by a nuclear medicine physician (MS) with blinding to the clinical and biological data. Data for each patient were analyzed semi-quantitatively and only one SUV_max_ value was recorded for each patient, corresponding to the highest SUV value for all vascular segments.

MRI images were evaluated for thickening and enhancement of the aortic wall on delayed enhancement images and for luminal narrowing and dilation. Aortic wall thickening was considered present with wall thickness ≥2 mm^19^. MRI reviewers were blinded to the clinical results at the time of MRI image interpretation.

In GCA patients, typical FDG joint uptake patterns in PMR was evaluated with a standardized 0-to-3 visual grading system: Grade 0: No uptake, Grade 1: Uptake < liver uptake, Grade 2: Uptake = liver uptake, Grade 3: Uptake > liver uptake^18^. PMR on FDG PET/MRI was considered as active in case of grade 3.

Three PET/MRI patterns were defined: (i) “inflammatory,” with positive PET (grade 3) and abnormal MRI (stenosis and/or wall thickening); (ii) “fibrous,” with negative PET (grade 1 or 2) and abnormal MRI (stenosis and/or wall thickening); and (iii) “normal,” with both negative PET and MRI. Some patients underwent several PET/MRI during the follow-up.

### Statistical analysis

Data are expressed as median [range] for continuous variables and number (%) for qualitative variables. Fisher’s exact test was used to analyze qualitative variables and the Mann-Whitney or Kruskall Willis test for continuous variables. Correlation was assessed by Spearman’s correlation coefficient. Patients disease activity, laboratory data and immunosuppressive therapies have been compared between different PET/MRI patterns.

P < 0.05 was considered statistically significant. Statistical analyses were carried out using GraphPad Prism v5.1 (GraphPad Software, San Diego, CA USA).

## Results

### Patient characteristics at the time of PET/MRI

The study included 13 patients (median age: 67 years [range: 23–87], 10 (77%) females) who underwent 18 PET/MRI scans (TA, n = 10 scans; GCA, n = 8 scans) at diagnosis (n = 4), relapse (n = 7), or during remission (n = 7). Median time from diagnosis to PET/MRI was 44 months [range: 0–222]: 14 [0–61] months for GCA and 86 [4–222] months for TA (p = 0.004). Eleven of 18 scans (61%; n = 6 GCA and n = 5 TA) were performed in patients receiving treatment with steroids (median dose: 30 mg/day [range: 3–240]).

Among the 18 PET/MRI scans, the pattern was inflammatory for eight (44%) and fibrous for three (17%); the other seven (39%) scans were normal. Patients with an inflammatory pattern had more vascular signs than those with a fibrous pattern (75% vs. 14%, respectively; p = 0.04), but had similar rates of constitutional symptoms (Table 1). CRP level appeared to be correlated with SUVmax values (r = 0.4; p = 0.06). Median CRP levels were significantly higher with inflammatory and normal patterns than with the fibrous pattern (25 mg [6–100] and 26 mg [0–250] vs. 0 mg, respectively; p = 0.01). All patients with an inflammatory pattern had active disease compared to 3/7 (43%) with a normal pattern (p = 0.03). Median SUVmax was higher with an inflammatory pattern than with a fibrous or normal pattern (4.85 [3–8.6] vs. 1.9 [1.8–2.1] and 2.2 [2–4.4], inflammatory vs. others; p = 0.002). CRP level and number of patients on steroids at 6 months were similar with all PET/MRI patterns, as was the rate of remission (Table 1).

**Table 1:** Characteristics of patients with Takayasu arteritis (TA) and giant cell arteritis (GCA) by inflammatory, fibrous, and normal PET/MRI patterns.

Disease and patient characteristics	Inflammatory (n = 8)	Fibrous (n = 3)	Normal (n = 7)	P value
TA (n = 10 scans)	5 (63%)	3 (100%)	2 (29%)	0.3
GCA (n = 8 scans)	3 (38%)	0	5 (62%)	0.3
Vascular signs	6 (75%)	0	1 (14%)**	**0.04**
Constitutional symptoms	5 (63%)	0	3 (43%)	0.6
C-reactive protein level (mg/L)	25 [6–100]*	0	26 [0–250]	**0.01**
Active disease	8 (100%)**	0 (0%)	3 (43%)	**0.03**
SUVmax	4.85 [3–8.6]**	1.9 [1.8–2.1]	2.2 [2–4.4]	**0.002**
Steroids	4 (50%)	1/3 (33%)	(71%)	0.2
Time from diagnosis to PET/MRI (years)	2.9 [0.3–19]	9 [8.7–10]	4.4 [0–15]	0.2
Remission at 6 months	4 (50%)	3 (100%)	6 (86%)	0.2
Steroids/other drugs at 6 months	6 (75%)/6 (75%)	0	5 (71%)/3 (43%)	0.3
C-reactive protein level (mg/L) at 6 months	6.5 [0–27]	0	10 [0–24]	0.4

Data shown are median [range], or n (%).

*p < 0.05 (Kruskall Willis), **p < 0.05 inflammatory vs. normal PET/MRI.

SUVmax, maximum standardized uptake value.

**Figure 1:**
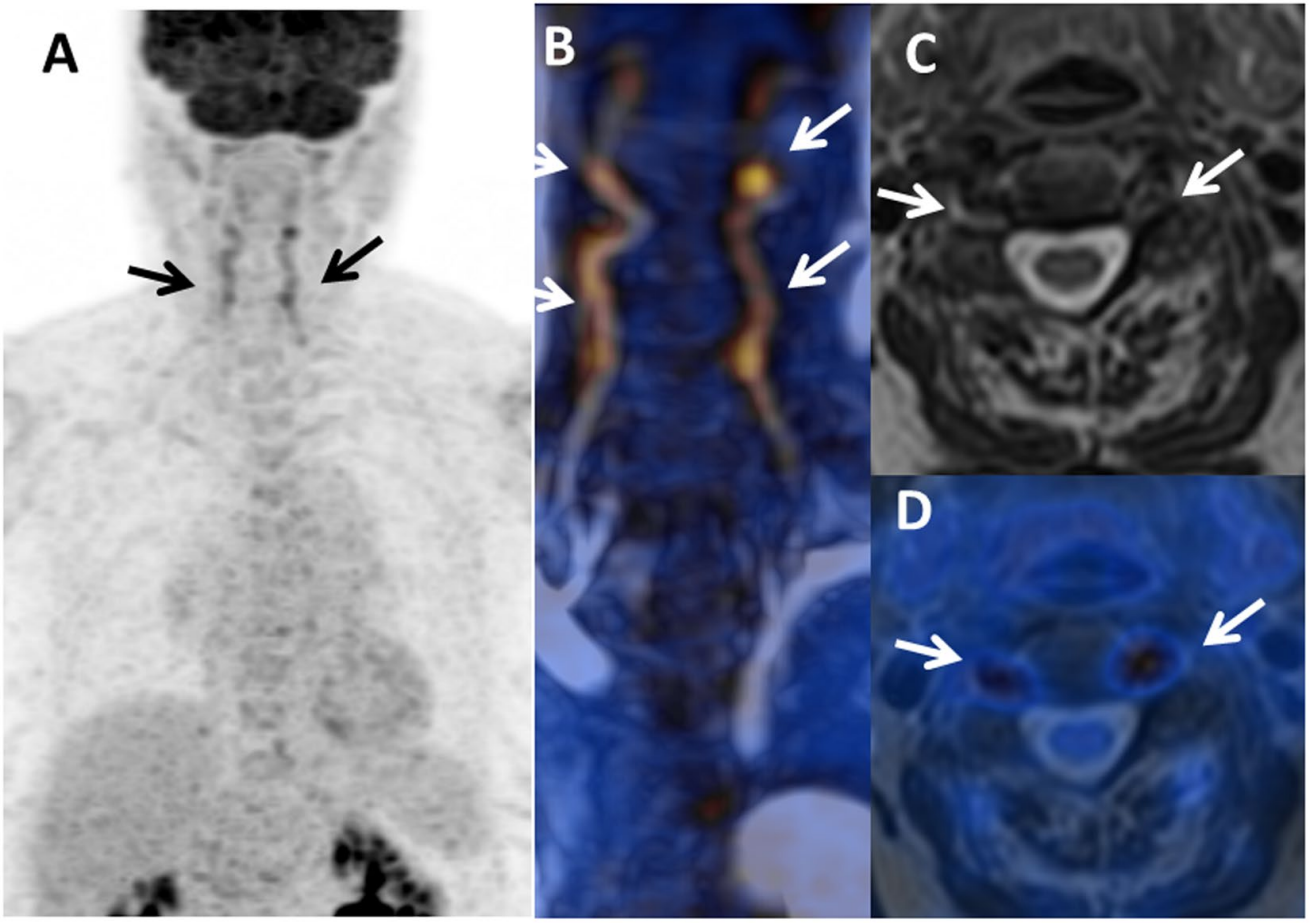
PET/MRI used for the initial diagnosis of giant cell arteritis (GCA) in a female with temporal headaches and acute-phase reactants without any vascular signs or arthralgia. PET/MRI showed an inflammatory pattern with clear uptake (>liver uptake, grade 3) in vertebral arteries (**(A)** Maximum intensity projection, and **(B)** fusion MR angiography/PET; arrows) associated with arterial wall thickening on: **(C)** MR axial T2-weighted image and **(D)** T2-weighted/PET fusion.

**Figure 2:**
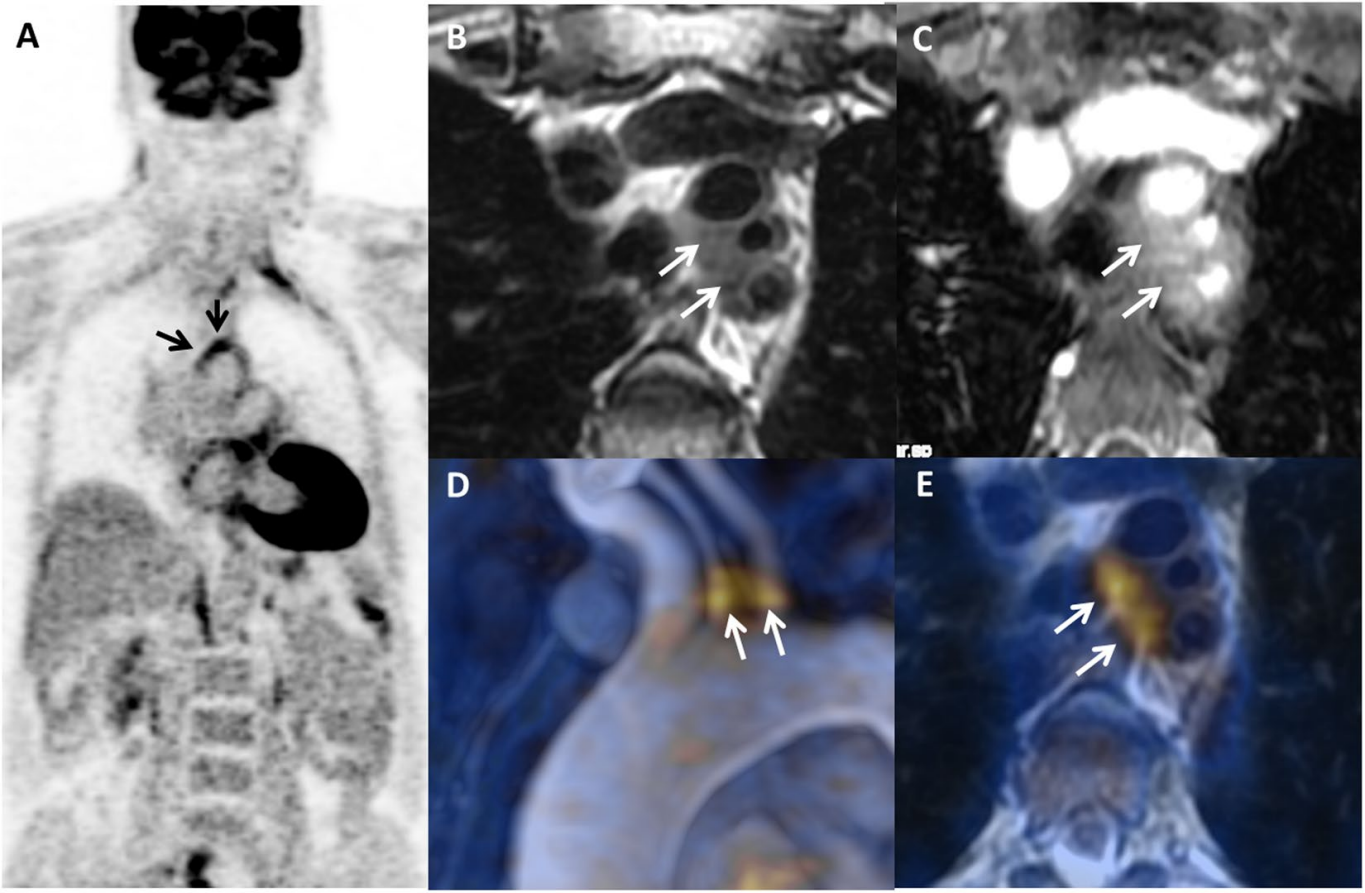
Case of Takayasu arteritis (TA) in a 45-year-old female with arthralgia, vascular claudication, and acute phase reactants refractory to steroids and methotrexate. PET/MRI ((A) coronla PET, **(B)** T2-weighted image, **(C)** post-contrast T1-weighted image, **(D)** fusion MR angiography/PET, **(E)** fusion PET/T2-weighted image) showed an inflammatory pattern with clear uptake (grade 3) at the origin of supra-aortic vessels associated with arterial wall thickening on T2-weighted image (**A**, arrows) and wall enhancement (**B**, arrows). Fusion images **(C**,**D)** show excellent co-registration of FDG uptake and MR findings.

When comparing the 10 PET/MRI scans performed in patients with TA (n = 6) to the eight scans performed in patients with GCA (n = 7), the inflammatory pattern was found in 5/5 (100%) patients with active TA and 3/6 (50%) with active GCA (Table 2). PET/MRI findings were normal in only 2/10 (20%) TA scans and 5/8 (62%) GCA scans (p = 0.3). Two GCA scans showed periarticular uptake compatible with polymyalgia rheumatica. Median SUVmax was similar in GCA and TA (3.4 [2.1–8.6] vs. 2.6 [1.8–7.1], respectively; p = 0.4). The frequency of vascular signs and constitutional symptoms, CRP level and prednisone dose (although not significantly) was higher in active disease (n = 11 scans) than in remission (n = 7 scans) (Table 3). Median SUVmax was 4.7 [2.1–8.6] vs. 2 [1.8–2.6] in patients with active disease vs. remission, respectively (p = 0.003); the inflammatory pattern was found in 8/11 (73%) scans from patients with active disease vs. none with remission, whereas a normal or fibrous pattern was found in 27% vs. approximately 50% of scans in patients with active disease vs. remission, respectively. Illustrative cases are shown in Figs 1 and 2. Among the GCA patients, no significant FDG uptake has been observed in temporal arteries.

**Table 2:** Characteristics of patients with TA and GCA at the time of PET/MRI.

	All (GCA + TA) (n = 13, 18 scans)	GCA (n = 7, 8 scans)	TA (n = 6, 10 scans)	P value (GCA vs TA)
Vascular signs	7/18 (39%)	3/8 (38%)	4/10 (40%)	0.7
Constitutional symptoms	8/18 (44%)	6/8 (75%)	2/10 (20%)	0.01
C-reactive protein level (mg/L)	23 [0–250]	25.5 [0–250]	7.5 [0–100]	0.2
PET/MRI inflammatory pattern	8/18 (44%)	3/8 (17%)	5/10 (50%)	0.6
PET/MRI fibrous pattern	3/18 (17%)	0/8 (0%)	3/10 (30%)	0.2
PET/MRI normal	7/18 (39%)	5/8 (62.5%)	2/10 (20%)	0.3
SUVmax	3.0 [1.8–8.6]	3.4 [2.1–8.6]	2.6 [1.8–7.1]	0.4
FDG uptake with polymyalgia rheumatica	2/18	2/8 (25%)	NA	—
MRI arterial thickening (>2 mm)	10 (61%)	2/8 (25%)	8/10 (80%)	0.05
MRI stenosis	6/18 (33%)	0/8	6/10 (60%)	0.01
Active disease (clinical and/or biological signs)	11/18 (61%)	6/8 (75%)	5/10 (50%)	0.4
Steroids at the time of PET/MRI	10/18 (56%)	6/8 (75%)	4/10 (40%)	0.4
Steroids (mg/day)	30 [3–240]	50 [15–240]	12.5 [3–45]	0.005
Time between steroid initiation and PET/MRI (months)	28 [0–518]	28 [0–518]	53 [0–121]	0.9
Time between diagnosis and PET/MRI (months)	44 [0–222]	14 [0–61]	86 [4–222]	0.004

Data are median [range], or n (%).

**Table 3:** Characteristics of patients with active disease and remission.

	Active disease (n = 11 scans)	Active GCA (n = 6 scans)	Active TA (n = 5 scans)	Remission (n = 7 scans)	P value (active vs. remission)
TA	5/11 (45%)	—	—	5/7 (71%)	0.2
GCA	6/11 (55%)	—	—	2/6 (33%)	0.2
Vascular signs	7/11 (64%)	3/6 (50%)	4/5 (80%)	0	**0.04**
Constitutional symptoms	8/11 (73%)	6/6 (100%)	2/5 (40%)	0	**0.001**
C-reactive protein level (mg/L)	25 [0–250]	25 (0–250)	26 (6–100)	0 [0–30]	**0.006**
PET + (grade ≥3)	9/11 (82%)	4/6 (66%)	5/5 (100%)	0	**0.003**
PET/MRI inflammatory pattern	8/11 (73%)	3/6 (50%)	5/5 (100%)	0	**0.004**
PET/MRI fibrous pattern	0	0	0	3/7 (43%)	**0.04**
PET/MRI normal	3/11 (27%)	3/6 (50%)	0	4/7 (57%)	0.3
SUVmax	4.7 [2.1–8.6]	3.4 (2.1–8.6)	4.7 (3–7.1)	2 [1.8–2.6]	**0.003**
Steroids	6/11 (55%)	4/6 (66%)	2/5 (40%)	4/7 (57%)	1
Prednisone dose (mg/day)	52.5 [15–240]	—	—	12.5 [3–40]	0.08
Time between diagnosis and PET (months)	25 [0–222]	—	—	138 [14–179]	0.2

Data are as median [range], or n (%).

## Discussion

This retrospective study describes the hybrid PET/MRI findings in 13 patients with LVV and relates them to clinical characteristics and outcome. PET/MRI analysis distinguished three different patterns and this classification agreed with the clinical setting. The inflammatory pattern, defined as both abnormal PET and MRI, was highly associated with disease activity, particularly in TA. This pattern was found in all scans from patients with active TA disease compared to only 50% of scans from patient with active GCA.

PET/MRI combines the quantitative measurement of radiotracer uptake with the multi-contrast anatomic assessment of MRI to offer a comprehensive “all-in one” vascular scan. In our study, we introduced new imaging criteria, integrating PET and MRI findings, and defined two imaging patterns to define LVV status. The inflammatory pattern suggests the presence of an inflammatory process, whereas the fibrous pattern suggests fibrotic lesions without “macroscopic” inflammation. Importantly, 43% of PET/MRI were normal, all in GCA patients and this could be explained by the prevalence of aortitis in GCA. At the difference of Takayasu disease with presence of aortitis in all cases, in GCA the aortitis is present in 30–60% of cases. The objective of this classification is to provide an “all-in-one” tool for clinicians to assist in the identification of disease activity and select a personalized therapeutic strategy. Clinical acceptance of PET/MRI is a challenge because it is necessary to identify clinical scenarios in which data from both PET and MRI are required, and to assess the impact of simultaneous PET/MRI acquisition on disease detection, characterization, and subsequent disease management.

In LVV imaging, combining different morphological parameters (wall thickening, enhancement) with biological parameters (FDG glucose metabolism) can assist in the characterization of disease status. Moreover, the simultaneous acquisition of PET and MRI data offers good co-localization of anatomic structures and the biological process occurring within these structures. MRI data would also be helpful in older patients, who often have atherosclerosis, because atherosclerotic plaques can accumulate 18F-FDG, leading to false-positive results^20^.

Finally, by increasing the sensitivity for the detection of vasculitis using digital PET detectors and integrated PET/MRI analysis, the use of invasive tests such as temporal artery biopsy may not be necessary. TAB is usually performed in GCA, but the estimated false-negative rate ranges from 6–17%, especially if the biopsy is obtained in an arteritis-free segment^21–25^. In a study of 120 patients with large-vessel GCA defined by radiographic evidence of subclavian artery vasculitis attributed to GCA, temporal artery biopsy findings were positive in only half of the cases^26^. A recent retrospective study have evaluated the diagnostic performance of 18 F-FDG PET-CT for large vessel involvement in patients with suspected giant cell arteritis and negative TAB^27^. In these 63 patients with negative TAB, 18F-FDG PET-CT showed large vessel involvement in 22 patients, 14 of whom were finally diagnosed with GCA and 41 patients were 18F-FDG PET-CT negative, 9 of whom were finally diagnosed with GCA.

Compared with PET/CT, PET/MRI can reduce the radiation dose to the patient, thus facilitating repeated use for patient if necessary, to evaluate the disease activity during follow-up. In GCA, repeated PET/MRI could help to confirm clinical relapse, and in TA, repeated PET/MRI could be used to determine remission and absence of arterial progression. Last EULAR recommendations in 2018^28^ propose to use MRI for these young patients instead of CT because of the need of regular follow-up imaging. Another important advantage of combined MRI can be the better analysis of vascular arterial gadolinium uptake, as well as concomitant heart analysis. Indeed, in GCA, repeated PET/MRI could help confirm clinical relapse, and in TA, repeated PET/MRI could be used to determine remission and absence of arterial progression. FDG has been successfully applied in inflammatory diseases since many years. However, in addition to glucose metabolism, a large variety of targets for inflammation imaging are being discovered and explored in several pre-clinical or clinical studies. For instance, radiotracers have been developed to explore membrane markers of inflammatory cells such as translocator protein (TSPO) or somatostatin receptor, inflammatory cytokines such as interleukin 2 or TNF-alpha, or also targets on inflammation related vessels such as integrin receptor. We believe that hybrid and multimodal imaging with tracers targeting different biomarkers will surely contribute to improved visualization and quantification of the vessel inflammation.

Our study presents several limits. One point is the use of only wall thickness on MR to positive MR scan for vasculitis. Other MR parameters, such as wall edema or contrast enhancement which have been previously analyzed in TA, have not been used in this small retrospective study. However, the pathological threshold when using this parameter is not consensual.

Another limit is that conventional 2D Black Blood sequence, a state of art MR sequence for vasculitis, was not used in this study. However, 2D Black Blood sequence are time-consuming, provide a limited scan area and cannot be reconstructed in various planes which is an issue in case of concentric wall thickening, hallmark of LVV. The recent feasibility study about the use of a T1w-3D black-blood turbo spin echo sequence for the diagnosis of thoracic LVV is promising, especially in the setting of hybrid PET/MR exploration^29^.

In conclusion, PET/MRI might be an alternative and comprehensive functional imaging approach for the assessment of LVV and could facilitate the characterization of disease activity, especially for challenging cases.

### Ethical approval

All procedures performed in studies involving human participants were in accordance with the ethical standards of the institutional and/or national research committee and with the 1964 Helsinki declaration and its later amendments or comparable ethical standards. All patients were enrolled prospectively in the “Promise” protocol (2015-A01431-48) and a written informed consent was obtained. This study was approved by national government authorities. (CPP Ile de France, Ambroise Paré, Paris France).
